# Dr. Moran on the Uses of the Oil of Turpentine

**Published:** 1830-01-01

**Authors:** 


### XXXI.

### ON THE USES OF THE OIL OF TURPENTINE.

This powerful remedy is in all probability not applied at present to many uses for which it is adapted. Its nauseousness and the disagreeable effects which it produces are great objections to its employment, but these may in some degree be got over by giving it in the form of emulsion in an aromatic water. At all events its actual powers are worth ascertaining by experiment, and on that account we are induced to notice a short paper by Dr. Moran, contained in a Journal for which we have to thank a friend :—The EDINBURGH MONTHLY EXAMINER.

"Having suffered several months," says Dr. Moran, "from an obstinate intermittent fever which would not yield to large doses of Bark or Sulphur, I was led often to reflect upon the cause and its removal of the various forms of fever, until, as I judged by the best and most concise reasoning, as above explained, I formed the plan of cure; therefore, as an expulsive and correcting evacuant, the Ol. Terebinthinæ, a medicine I have been in the habit of employing in the cure of many diseases for several years past, an account of which I shall give in the sequel, struck me as being likely to produce a good effect.

"It was double tertian, and the paroxysm, I found about 7 o'clock in the evening, to be coming on. I mixed 2 ounces of the Ol. Terebinthinæ well in sugar and water, and drank the whole at once,—it created a warm glow in a short time, first in the stomach, and afterwards generally over the frame. I by no means found it so unpleasant as I had expected; I had to observe a reclined and quiet posture to prevent the stomach from rejecting it, which more than once nearly happened, in the course of 20 minutes from the time of taking it; I perceived the violet odour in my urine, and from the moment I had taken it, all symptoms of the paroxysm disappeared, and from that time, ceased altogether. In about an hour, it produced violent Catharsis, which lasted for several hours, and micturition attended with neither stranguary or uneasiness in the act; the violet smell continued for eight days in the urine, shewing how permanent and diffusive its action must have been; after this, I treated other cases of Intermittents and other fevers with the same remedy, with decided advantage, the only precaution I think necessary in its exhibition is, that it must not be given unless the stomach be empty, nothing to be taken after it until the Catharsis has commenced, when some whey, tea, or warm broth may be plentifully used; if taken on a full stomach, or if food be taken after it, it violently attacks the head, and produces a state like Epilepsy, which I twice witnessed, but relieved each by causing the patients to empty the stomach, after which they felt quite well; its dose may be from half an ounce to one ounce mixed with honey or sugar.

"From the trials I made with it, I can recommend it as a safe and very useful medicine in fevers of all descriptions, but I by no means wish it to be understood that it is to do away with blood-letting whenever required; on the contrary. I in all cases when necessary, recommend venesection beforehand, but I am strongly of opinion that its use may cause many of the medicines used in fever to be dispensed with.

"From what I have above said, it will follow of course, that I recommend the use of Ol. Terebinthinæ in the plague, which I do most confidently, as a medicine capable of removing, correcting, or controlling the cause of the disease; but it should in all cases be had recourse to, at the commencement, before habit be confirmed, the vital powers much diminished, or functions destroyed; by a steady and careful exhibition of it in febrile or other diseases, joined with blood-letting when indicated, I have no doubt by recommending it, I shall have the heartfelt satisfaction to find that I have conferred a benefit on mankind, and that I have contributed my mite at least to the alleviation of much human suffering.

"Among the diseases of the order Phlegmasiæ, I treated, but Enteritis, Peritonitis, Rheumatismus, and Podagra, with the Ol. Terebinthinæ; but had I an opportunity, I think it could be employed with great advantage, in Hepatitis, both acute and chronic, as also in several others of the order. In Enteritis and Peritonitis, I premised a Bleeding, and gave an ounce and a half mixed as before, after which the symptoms all quickly subsided; for its use in this disease, the public are indebted to the eccentric but clever Doctor Brennan of Dublin, who pointed it out many years ago as of the greatest use in puerperal fever, who is richly entitled to the thanks, at least of his countrywomen, for so valuable a remedy.

"In Rheumatisms both acute and chronic, I found it, after having premised a bleeding, to act in the most satisfactory manner, and remove the disease. I was myself seized with inflammation of the great toe as I first judged it to be, for which I tried all the usual remedies, such as poulticing, local bleeding, blistering, &c., but all to no purpose for several days, when I discovered that it was an attack of gout; the fever I found came on every evening, and lasted until morning, when, after having writhed in agony all night, I obtained some repose, and continued free from pain all the next day, and in other respects well except the pain, and inability to use the feet. I soon perceived the similarity there was between the fever that accompanied it, and the obstinate intermittent I before made mention of, I therefore had recourse to the remedy that had so quickly removed it, in the same dose, and I had the pleasure to find that it was equally efficacious, the fever from that moment entirely ceased, and in a few days I was as well as ever.

"I was twice afterwards threatened with an attack of gout, but a dose of the medicine as often prevented it; in the latter case, I was seized in the night, and in the morning I could not use the left foot or leg; to put it to the ground was a thing impossible, I sent for a dose of the medicine, which I took, and had the part well rubbed with some of the same; in about three hours afterwards, or after the medicine had had an effect, I was well, able to walk about, and travelled in a boat all night, and continued up to the present time, without any symptom of it, though my manner of living be the same. I feel so confident of being able to prevent its attack, that I by no means feel the least alarm at any inconvenience from gout; but also to prevent it, I strongly recommend bathing the feet night and morning in cold water, and never to place them in warm, paying attention always to the state of digestion, &c. of the bowels.

"Since then, I have had an opportunity but once of trying its effect, and that was on a woman of 50 years of

-age, with irregular gout, upon whom the medicine had the desired effect, and soon put an end to her suffering, after which she enjoyed much better health than for many years before. In Hysteria and Hysteritis, I have every reason to think its use would be attended with great benefit to the sufferers, but I have had no opportunity of trying it.

"In Hæmorrhois I have used the Ol. Terebinthinæ with success, and also in internal Hæmorrhage from the stomach, I am inclined to think it would be useful in Hæmorrhois petechialis.

"In Catarrhus, both acute and chronic, after having premised a full bleeding, I used it repeatedly with great success, the symptoms having all quickly subsided after its use.

"In Dysentery, which is but a Catarrhus of the mucous membrane of the intestines, I used it also with success, but I gave Calomel in doses of 10 grains with it, together with fomentations, bleeding, and such treatment as the severity of the symptoms required.

"In Apoplexy, I have had no opportunity of using it, although I think great benefit might be derived from it in that disease.

"In Paralysis, I found it a valuable remedy, used as an embrocation, a cathartic, and also mixed with honey in small doses as an alterative. In this disease, I first witnessed its exhibition in Jervis Street Hospital, Dublin, when a Student under Dr. Francis Brooks, whose suavity of manner alike engaged the confidence of the patient, and the attention of the pupil.

"In Dyspepsia, Hypochondriasis and Chlorosis, I mean to use it whenever an opportunity offers.

"In Cholera Morbus I have used it with great advantage in many cases of the very worst description, in the following manner.—The moment the patient was admitted he received 10 grains of Calomel, and the Ol. Terebinthinæ mixture; I then had him placed in a hot bath, and well rubbed while in the bath, all over the body,—after 20 or 30 minutes he was taken out, well rubbed with flannels, and carefully laid in bed between hot blankets. After which 100 drops of the Tincture of Opium in Camphor mixture was given; he very soon was thrown into a copious perspiration, followed by sleep, after which the spasms and vomiting gradually subsided. The following morning 5 grains of Calomel exhibited, and 60 drops of Tincture of Opium at night entirely removed the disease—a second dose of the medicine was rarely required. I have had many cases brought into Hospital more resembling dead than living beings, the debility and exhaustion was so great, yet from cases such as those I was occasionally obliged to extract blood. After the bath the reaction was so great that I found the use of the lancet indispensable; and here I would advise in all cases after reaction has set in, when the face and neck assume a crimson colour, or a colour approaching to rose pink, that blood be extracted without loss of time, otherwise the loss of life will be almost certain. I am thus led to advise from experience, for at first I was much averse to bleeding in the disease, until I met with such a case as the above, in which I withheld the use of the lancet, but the patient died, and the appearances on dissection, shewed me that he died from extravasation at the base of the brain, congestion in the vessels, and effusion into the ventricles and between the membranes. After this, in all cases of that nature I have used the lancet with the most decided advantage, never having afterwards lost a case. This practice I hope may prove a benefit to our suffering soldiers in India, at all events I strongly recommend its adoption to the board of Directors, and if it prove as successful as I have found it, it will be no small degree of comfort to me to have pointed it out. To Sir James M'Grigor too, who is ever alive to promote whatever may tend to remove disease, and who has already laboured to that purpose with so much effect, I very respectfully suggest its adoption by those under his controul, in whose hands, if it prove of benefit to the soldiery, I shall have a soldier's feeling, that of having done my duty.

"In Hydrophobia I would suggest its use by general frictions and internal exhibition, joined with Calomel and Tincture of Opium.

"In Anasarca and Ascites, I found it of great use given with the Squill Pill and Calomel. In recent cases and young people I have not found it fail in removing the diseases.

"In Jaundice I found it an excellent remedy, together with Calomel and Hot Bath.

"In cases of Worms of all kinds, whether in children or adults it may be given with safety and advantage, in doses from one drachm to two drachms; mixed with Honey and water.

"In cases of Snake bite, I gave it with marked success. The recital of one may be necessary to shew the treatment pursued, viz :—

"A man was brought to the Hospital, who had been bitten by a black Snake, about half an hour before, or such time as enabled him to arrive in a quick pace a distance of a mile. The bite was situate over the radial artery of the right arm, to which the Snake had clung for several seconds so firmly, that shaking the arm strongly did not remove him, until removed by another person. When I saw him the greatest terror and anxiety were marked in his countenance, which was also red and tumid, the eyes dull and heavy, and the adnata suffused with red vessels. He complained of a violent desire to sleep, and expressed himself that he would give the world to be permitted to lie down. I gave an oz. of the Ol. Terebinthinæ in a mixture with sugar and water, and had the arm bound with a ligature above the elbow; I cut out as much of the bitten part as I could with safety in the situation it was in, and had the wound rubbed with liq. Carb. *Ammoniæ*, then the whole arm was immersed in hot water, and rubbed down between the hands from the place where the ligature, was applied to the wound. After this I extracted 2lbs of blood from the arm, and ordered two drachms of the Ol. Terebinthinæ to be given every quarter of an hour, until it produced a Cathartic effect. The great desire to sleep continued for several hours, but the patient was kept awake by being made to walk about supported by two men. The medicine began to operate in about an hour, and continued to do so for two hours, after which the head became relieved. In eight hours after admission, he was permitted to sleep, after which he awoke without uneasiness, his mind being perfectly cheerful and composed. Several other cases were treated in the same way with similar results, but in neither of them was this kind of Snake seen, it therefore cannot be so much relied on as the first, in which the Snake was seen for several seconds hanging on the arm by the teeth."

We need scarcely say that, however, we may be disposed to admire the good feeling and honest enthusiasm of the author, we cannot go along with him in all his speculations and all his hopes. Cholera, alas! and the plague will ravage and destroy, when the oil of turpentine and its advocate shall perhaps have been forgotten. Neither are rheumatism and the gout to be banished from the nosologic chart or the human frame, at least whilst man shall suffer from cold or enjoy good living. At the same time the foregoing observations on the medicine, when taken cum grano, are interesting, and may furnish a hint or two to those who have wit enough to discriminate what is fact from what is fancy. We need scarcely say that we devoutly believe all *our* readers are of this description.

---

